# Integrated isotope-assisted metabolomics and ^13^C metabolic flux analysis reveals metabolic flux redistribution for high glucoamylase production by *Aspergillus niger*

**DOI:** 10.1186/s12934-015-0329-y

**Published:** 2015-09-17

**Authors:** Hongzhong Lu, Xiaoyun Liu, Mingzhi Huang, Jianye Xia, Ju Chu, Yingping Zhuang, Siliang Zhang, Henk Noorman

**Affiliations:** State Key Laboratory of Bioreactor Engineering, East China University of Science and Technology, 200237 Shanghai, People’s Republic of China; DSM Biotechnology Center, P.O. Box1, 2600 MA Delft, The Netherlands

**Keywords:** *Aspergillus niger*, Glucoamylase, ^13^C metabolic flux analysis, Metabolomics, Cofactor metabolism

## Abstract

**Background:**

*Aspergillus niger* is widely used for enzyme production and achievement of high enzyme production depends on the comprehensive understanding of cell’s metabolic regulation mechanisms.

**Results:**

In this paper, we investigate the metabolic differences and regulation mechanisms between a high glucoamylase-producing strain *A. niger* DS03043 and its wild-type parent strain *A. niger* CBS513.88 via an integrated isotope-assisted metabolomics and ^13^C metabolic flux analysis approach. We found that *A. niger* DS03043 had higher cell growth, glucose uptake, and glucoamylase production rates but lower oxalic acid and citric acid secretion rates. In response to above phenotype changes, *A. niger* DS03043 was characterized by an increased carbon flux directed to the oxidative pentose phosphate pathway in contrast to reduced flux through TCA cycle, which were confirmed by consistent changes in pool sizes of metabolites. A higher ratio of ATP over AMP in the high producing strain might contribute to the increase in the PP pathway flux as glucosephosphate isomerase was inhibited at higher ATP concentrations. *A. niger* CBS513.88, however, was in a higher redox state due to the imbalance of NADH regeneration and consumption, resulting in the secretion of oxalic acid and citric acid, as well as the accumulation of intracellular OAA and PEP, which may in turn result in the decrease in the glucose uptake rate.

**Conclusions:**

The application of integrated metabolomics and ^13^C metabolic flux analysis highlights the regulation mechanisms of energy and redox metabolism on flux redistribution in *A. niger*.

**Graphical abstract:**
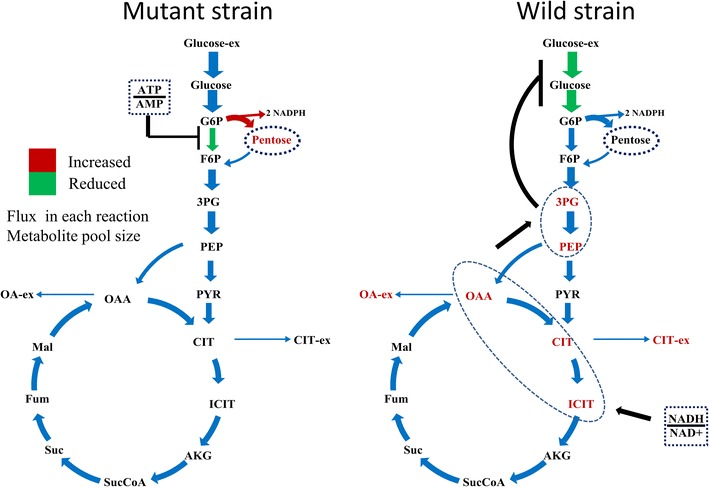
An integrated isotope-assisted metabolomics and ^13^C metabolic flux analysis was was firstly systematically performed in *A. niger*. In response to enzyme production, the metabolic flux in *A. niger* DS03043 (high-producing) was redistributed, characterized by an increased carbon flux directed to the oxidative pentose phosphate pathway as well as an increased pool size of pentose. The consistency in ^13^C metabolic flux analysis and metabolites quantification indicated that an imbalance of NADH formation and consumption led to the accumulation and secretion of organic acids in *A. niger* CBS513.88 (wild-type)

**Electronic supplementary material:**

The online version of this article (doi:10.1186/s12934-015-0329-y) contains supplementary material, which is available to authorized users.

## Background

*Aspergillus niger*, one of the most important filamentous fungi, has been widely used for the production of organic acids [1–3], such as citric and gluconic acid, and for the production of enzymes [4, 5] including glucoamylase, proteases, cellulase and glucose oxidase due to its excellent capacity in expression and secretion of proteins.

Despite the wide usage of *A. niger* for enzyme production, metabolic regulation mechanisms for high enzyme production by *A. niger* are still largely unclear. The complexity in metabolic regulation for high enzyme production calls for the introduction of multi-omics analysis. Metabolomics and fluxomics analysis, as an important complement to the commonly used genomics, transcriptomics and proteomics analysis, have undergone a quick development during the past years [6, 7]. With the aid of metabolomics analysis, the bottleneck for the target product synthesis can be identified [8], thus the addition of the pivotal limiting precursors can largely improve the strain performance. In the perturbed experiments, the profiles in dynamic changes of metabolites pool sizes can be used to assess the kinetic information of intracellular enzymes and finally estimate the limiting steps for the cell metabolism [9, 10]. Furthermore, the metabolomics play an important role in revealing the effects of energy metabolism and accumulation of polysaccharides on the strain physiology [11].

^13^C metabolic flux analysis has become a powerful tool to study the strain metabolic properties due to its capability of providing precise in vivo flux data [12–14]. For fructofuranosidase production by *A. niger*, Driouch et al. [15] reported that the production of recombinant enzyme led to a flux redistribution and in the recombinant strain the cytosolic pentose phosphate pathway (PPP) and mitochondrial malic enzyme was activated, indicating that the supply of NADPH is essential for efficient production of this protein. Klein et al. [16] observed that there is a positive relation between the lipid content and protein secretion by *Schizosaccharomyces pombe*. The latest ^13^C metabolic flux analysis also revealed that the imbalance in regeneration and utilization of cofactors result in the secretion of by-products [17].

To comprehensively understand the metabolic regulation mechanism for high glucoamylase production by *A. niger*, an integrated isotope-assisted metabolomics and ^13^C metabolic flux analysis was performed in this work. The integrative analysis of ^13^C MFA and metabolomics made then possible to identify the metabolic regulation mechanisms for high glucoamylase production by *A. niger* from the clear distinctions between the two strains in energy and redox metabolism.

## Results

### Physiology of *A. niger* DS03043 and CBS513.88

**Fig. 1:**
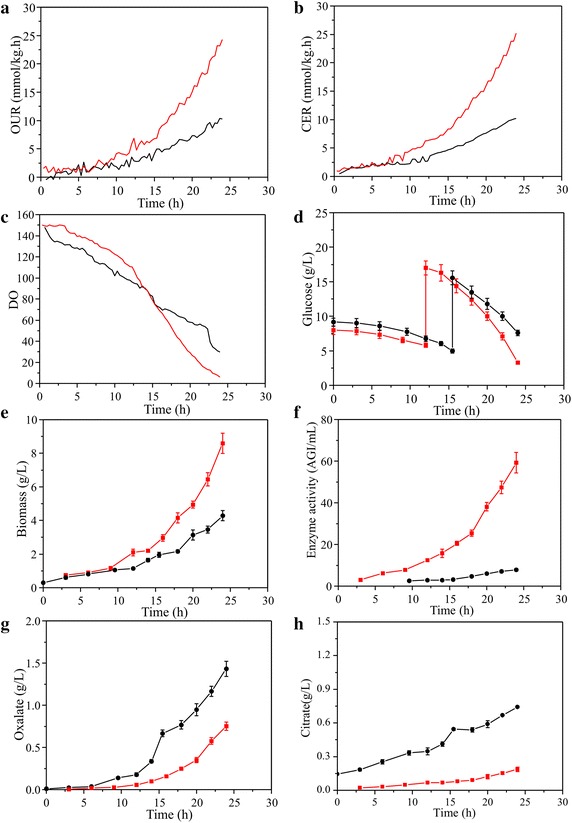
Physiological profiles of *A. niger* DS03043 (*red line*) and CBS513.88 (*black line*) in batch cultivations. The parameters including OUR (**a**), CER (**b**), DO (**c**), glucose concentration (**d**), biomass (**e**), enzyme activity (**f**), oxalate (**g**) and citrate (**h**) for. All parameters were measured based on at least triplicate measurements. The suddenly increased glucose concentration in **d** represent the pulsed addition of glucose during exponential phase

The two strains showed obviously different physiological characteristics (Fig. 1; Table 1). Consistent with the copy number of glucoamylase *gla*A gene, the specific glucoamylase production rate in *A. niger* DS03043 was about 8 times of that in *A. niger* CBS513.88. Whereas, the formation rate of oxalic and citric acid, as two main by-products, decreased significantly in *A. niger* DS03043. Though the specific growth and glucose uptake rate of *A. niger* DS03043 was about 40 and 25 % higher than those of *A. niger* CBS513.88, respectively, the two strains showed no statistically significant differences in the yields of biomass (Y_X/S_) and CO2 (Y_CO2/S_), initially indicating that mainly the reduction in by-products formation was compensated for the large increase in glucoamylase production by *A. niger* DS03043.

**Table 1:** Kinetic parameters and carbon recovery for *A. niger* DS03043 and CBS513.88

	*A. niger* DS03043	*A. niger* CBS513.88
A		
μ (h^−1^)	0.14 ± 0.01	0.10 ± 0.01
q_S_ (mmol/gDCW h)	1.83 ± 0.01	1.46 ± 0.01
q_GA_ (μmol/gDCW h)^a^	0.40 ± 0.03	0.05 ± 0.00
q_CIT_ (mmol/gDCW h)	0.02 ± 0.00	0.11 ± 0.01
q_OA_ (mmol/gDCW h)	0.11 ± 0.01	0.35 ± 0.02
q_O2_ (mmol/gDCW h)	2.94 ± 0.03	2.67 ± 0.02
q_CO2_ (mmol/gDCW h)	3.21 ± 0.02	2.79 ± 0.05
B		
Y_X/S_ (mmol/mmol)	0.49 ± 0.03	0.47 ± 0.02
Y_GA/S_ (mmol/mmol)	0.12 ± 0.01	0.02 ± 0.00
Y_CIT/S_ (mmol/mmol)	0.01 ± 0.00	0.07 ± 0.01
Y_OA/S_ (mmol/mmol)	0.02 ± 0.00	0.07 ± 0.01
Y_CO2/S_ (mmol/mmol)	0.30 ± 0.02	0.32 ± 0.03
Carbon recovery	94 %	95 %

All parameters were measured based on at least triplicate measurements. The closeness to 100 % of carbon recovery indicates that almost all carbon compounds produced have been considered

^a^q_GA_: Specific production rate of glucoamylase. For the calculation of q_GA_, 1 mg glucoamylase equals to 50 units of enzyme activity

### Estimation of intracellular metabolic flux and metabolites pool sizes

**Fig. 2:**
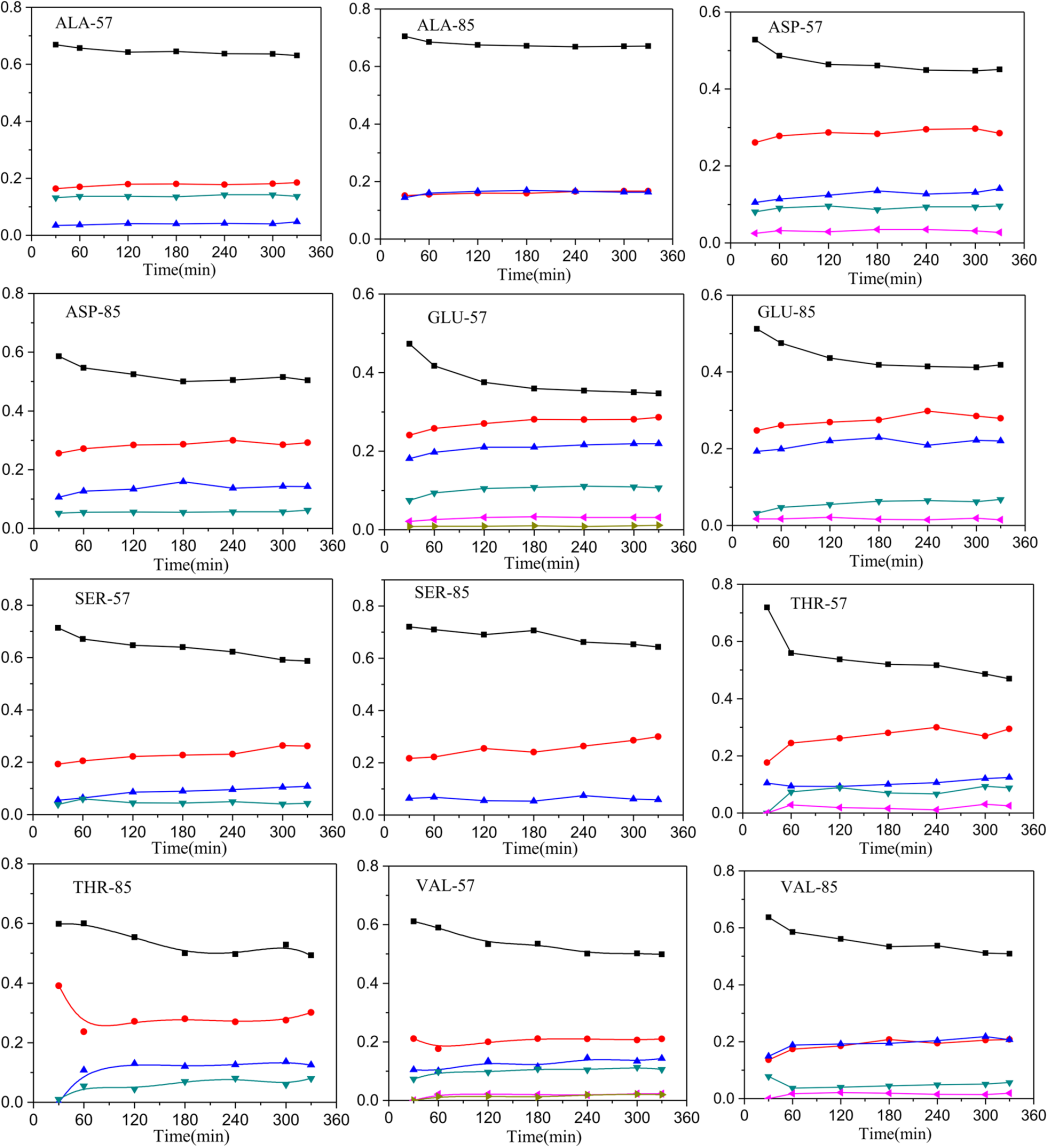
Profiles of enrichment for amino acid fragments in *A. niger* CBS513.88. The amino acid fragments including ALA-57, ALA-85, ASP-57, ASP-85, GLU-57, GLU-85, SER-57, SER-85, THR-57, THR-85, VAL-57 and VAL-85 after the labeled carbon source was fed into the broth

**Fig. 3:**
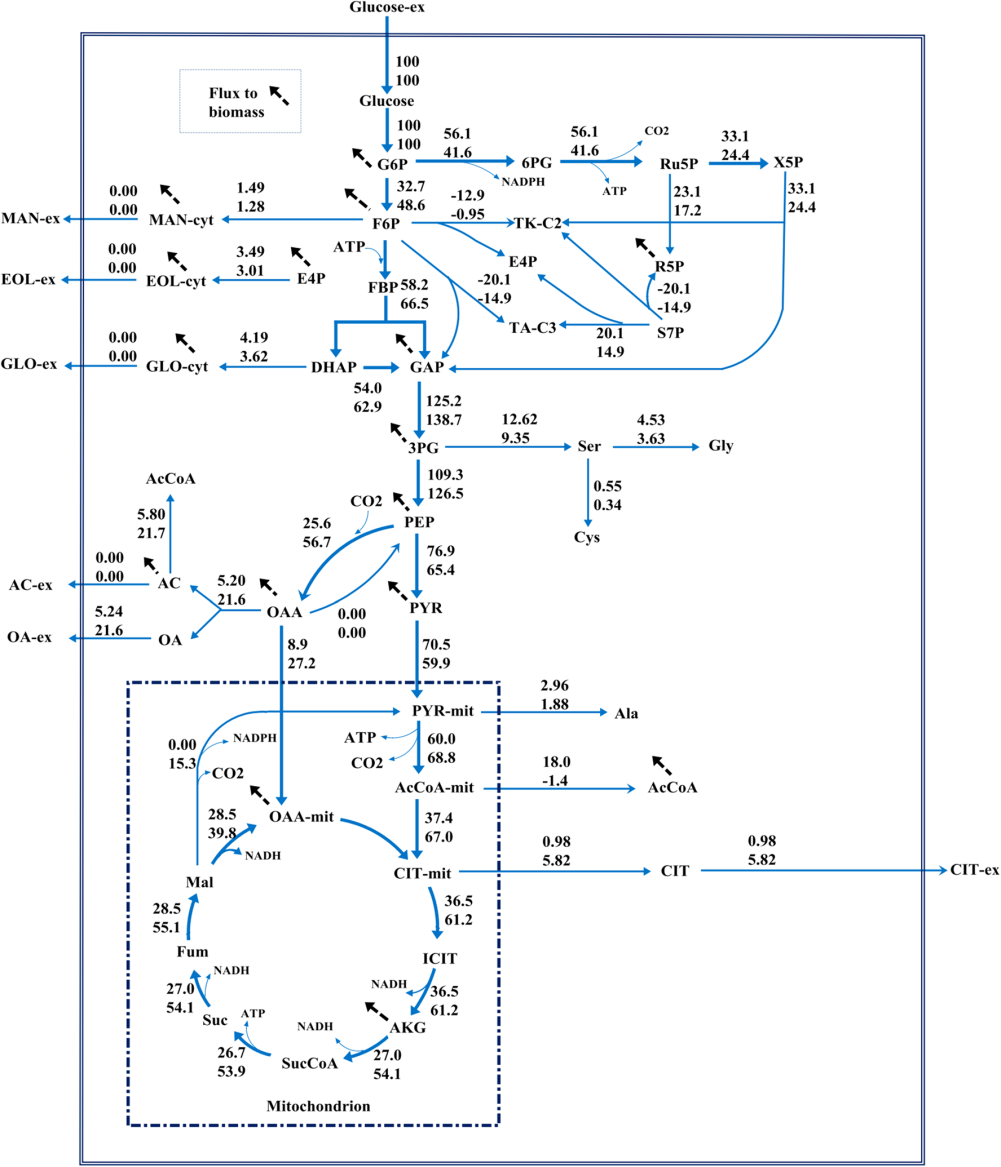
Relative flux (mmol/100 mmol glucose) of *A. niger* DS03043 (*upper*) and CBS513.88 (*lower*). The 68 and 95 % confidence intervals for each reaction and exchange flux values for reversible reactions can be found in Additional file 2. The detailed metabolic model of *A. niger* and the abbreviation of metabolites could be found in Additional file 4

The achievement of metabolic and isotopic pseudo steady-state is a pre-requisite for stationary ^13^C metabolic flux analysis. During batch cultivations, the specific growth rate and yield coefficients were approximately similar (data not shown), indicating that the strains were in metabolic steady-state. The labelling information for most of the free amino acids had reached the isotopic steady-state at 3 h after the labeled carbon was added into the broth (Fig. 2). Based on the kinetic parameters (Table 1) and the labelling information of free amino acids (Additional file 1), the fluxes were estimated using INCA software [18]. The best-fit flux distribution is shown in Fig. 3. The best-fit fluxes and confidence intervals are presented in Additional file 2. The obtained minimal weighted residual from the parameter estimations is below the cut-off value determined form the χ^2^ at a 95 % confidence level (Additional file 2), indicating that the estimated fluxes are statistically acceptable. The measured and estimated labelling information of amino acids fragments for optimal flux distributions is listed in Additional file 1.

**Fig. 4:**
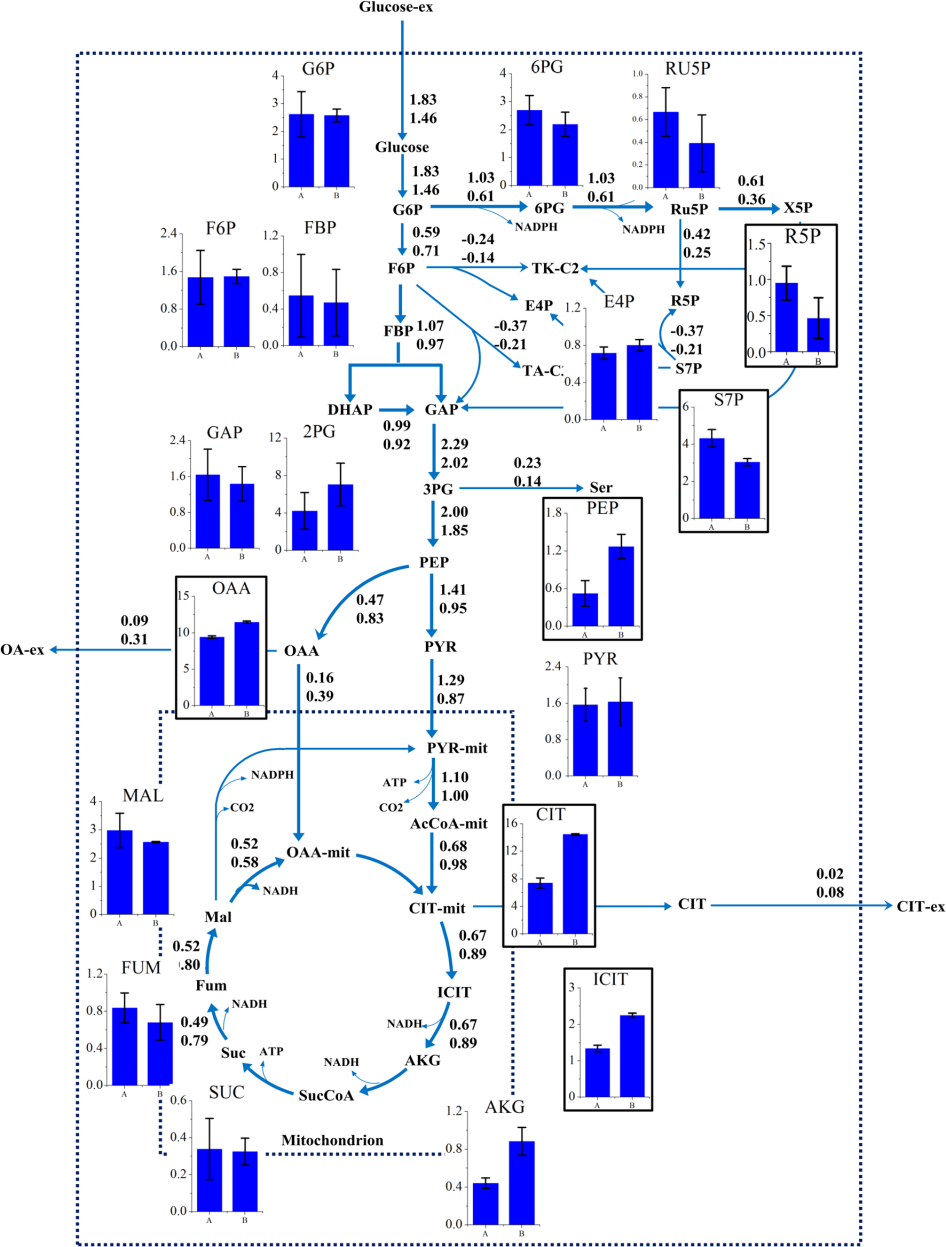
The distribution of metabolites pools between *A. niger* DS03043 (*left column*) and CBS513.88 (*right column*). The *bar* graphs with *black frames* indicate the significant difference (P value <0.05) in pool size of metabolites for the two strains. All metabolite pool sizes were measured in at least triplicate measurements. The P values were obtained from two-tailed T test statistical analyses. The *upper* and *lower numbers* represent the absolute flux values (mmol/gDCW h) through each reactions in *A. niger* DS03043 and CBS513.88 respectively

For both strains, the pool sizes of central carbon metabolites, amino acids and cofactors were determined by isotope-assisted LC–MS and EnzyFluo™ Assay Kit (Fig. 4; Tables 2, 3). In the following, an integrated isotope-assisted metabolomics and ^13^C metabolic flux analysis of central metabolism of *A. niger* was conducted to comprehensively understand the metabolic regulation mechanisms for high glucoamylase production by *A. niger*.

**Table 2:** Pool sizes of amino acids for *A. niger* DS03043 and CBS513.88

Name	*A. niger DS03043*		*A. niger CBS513.88*		P value of T test
	Value	SD	Value	SD	
Gln	56.23	3.05	51.20	4.81	0.73
Lys	52.36	3.88	46.50	6.50	0.66
Glu	63.85	3.71	44.00	4.68	0.01
Ala	25.56	1.39	13.74	2.36	6.36E−03
Asp	21.55	2.64	12.47	1.89	0.02
Thr	5.49	0.28	8.82	0.24	7.57E−05
Asn	6.94	0.45	8.61	0.25	4.45E−03
Trp	5.45	0.44	6.24	0.97	0.19
Orn	3.98	0.44	5.53	0.51	0.01
Tyr	3.63	0.18	5.36	0.94	0.07
Ser	5.78	0.41	5.03	0.70	0.43
Aaa	5.49	0.71	4.80	0.63	0.52
Val	2.46	0.07	3.54	0.43	0.04
Arg	4.11	0.33	3.44	0.34	0.24
His	2.07	0.08	1.61	0.19	0.62
Ile	0.86	0.03	0.98	0.13	0.19
Leu	0.72	0.01	0.87	0.14	0.14
Pro	0.84	0.07	0.58	0.09	0.04
Phe	0.64	0.02	0.44	0.08	0.06
Met	0.35	0.05	0.38	0.08	0.56

The unit is µmol/gDCW. All metabolite pool sizes were measured based on at least triplicate measurements. The P values were obtained based on two-tailed T test statistical analyses

**Table 3:** Pool sizes of nucleotides and coenzymes in *A. niger* DS03043 and CBS513.88 during batch cultivations

	*A. niger DS03043*		*A. niger CBS513.88*	
	Value	SD	Value	SD
GSH	0.46	0.01	0.31	0.03
GSSG	2.59	0.02	1.61	0.05
UTP	1.62	0.29	2.78	0.56
GTP	1.27	0.43	0.79	0.10
GDP	0.57	0.05	0.62	0.03
GMP	0.38	0.02	0.46	0.01
CMP	0.10	0.02	0.13	0.02
UMP	0.06	0.04	0.08	0.01
IMP	0.13	0.07	0.14	0.01
IDP	0.36	0.05	0.39	0.01
NADH	1.12	0.23	0.86	0.17
NAD^+^	10.31	0.72	5.33	0.70
NADPH	1.19	0.26	1.39	0.21
NADP^+^	5.13	0.51	4.55	0.96
ATP	13.37	1.67	6.68	0.81
ADP	1.39	0.36	1.17	0.27
AMP	1.29	0.39	1.20	0.45

The unit is µmol/gDCW. All metabolites pool size was measured at least triplicate measurements

### Central carbon metabolism

The central carbon metabolism, including EMP, PP and TCA cycle pathways, exhibited obvious differences between two strains. Firstly, consistent with a larger specific glucose uptake rate, most of the absolute fluxes in *A. niger* DS03043 were relatively higher than those of *A. niger* CBS513.88 (Fig. 4). In the following comparison, all fluxes are normalized to the specific glucose uptake rate (Fig. 3). The relative flux channeled into the PP pathway was obviously larger in *A. niger* DS03043 than in *A. niger* CBS513.88. Accordingly, the flux through EMP in *A. niger* DS03043 was decreased. Exceeding the demand for biomass synthesis, a fraction of pentose was recycled back into the EMP pathway in the form of F6P and GAP. As the main pathway for NADH formation, the flux through TCA cycle in *A. niger* DS03043 was only half of that in wild type strain (Fig. 3). Such results agree with previous studies involving *Aspergilli* [15, 19] and the detailed comparison could be found in Additional file 3.

The pool sizes of metabolites from the core carbon metabolic network showed a relationship with the flux distribution for both strains. Due to the increased flux from G6P to 6PG, the pool sizes of R5P and S7P in *A. niger* DS03043 were clearly larger (P value <0.05) than those in *A. niger* CBS513.88 (Fig. 4). In the upper EMP pathway, the pool sizes of G6P and F6P were nearly constant for the two strains, indicating that their pool sizes were strictly controlled. However, in the lower EMP pathway, except for PYR, the pool sizes of 3PG and PEP were largely increased in *A. niger* CBS513.88 (P value <0.05). It was also found that the intracellular OAA, CIT, isCIT and AKG pool sizes were obviously bigger in *A. niger* CBS513.88 than those in *A. niger* DS03043 (P value <0.05). On the contrary, in the TCA cycle of CBS513.88, the pool sizes of MAL and FUM were slightly smaller than *A. niger* DS03043. It could be speculated that there exists a close relationship between the accumulation of intermediates in the TCA cycle and the excretion of large amounts of oxalic acid and citric acid in *A. niger* CBS513.88.

### Amino acids metabolism

**Fig. 5:**
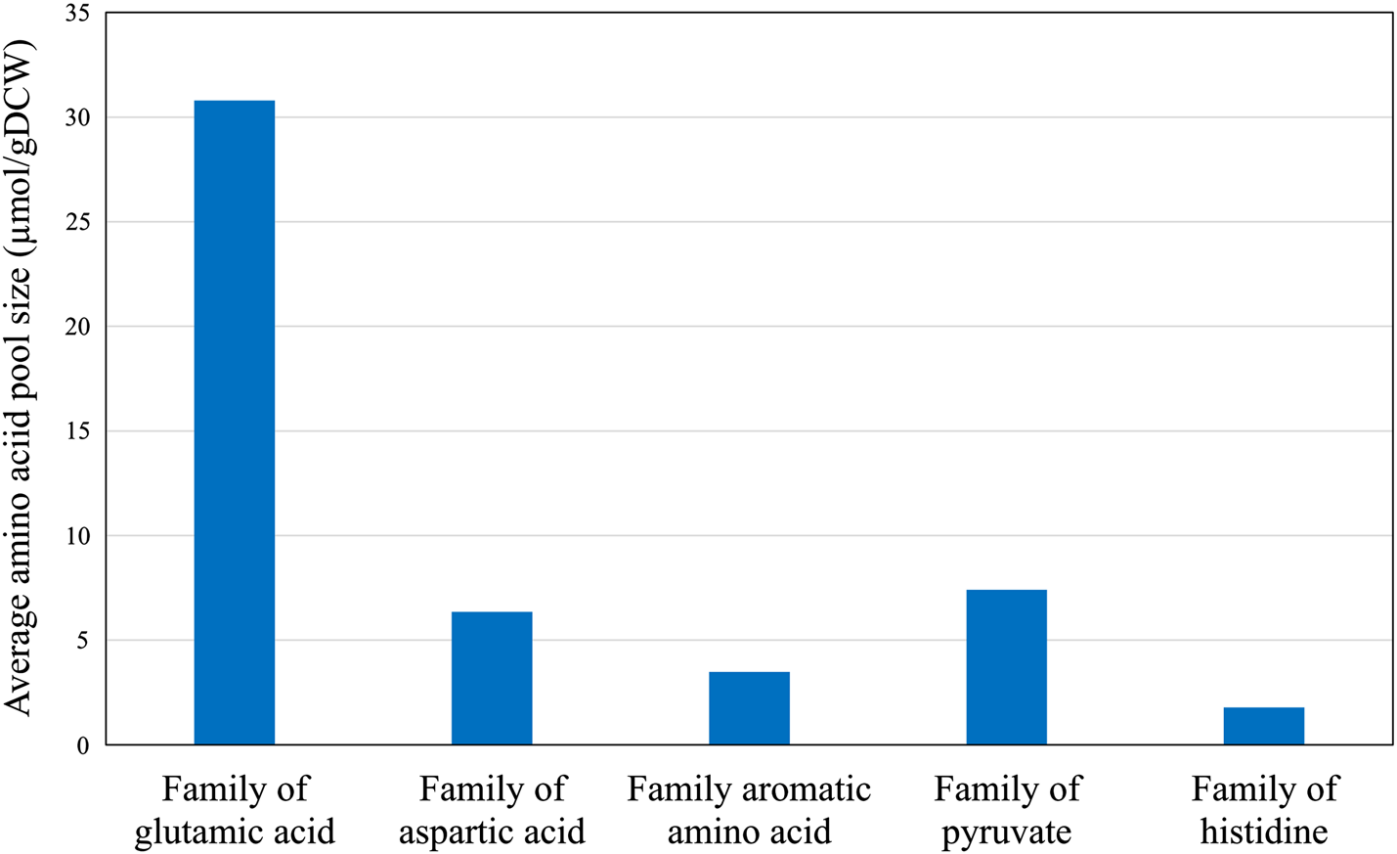
The average values of pool size for amino acids from different amino acid families

The amino acids pool sizes for *A. niger* DS03043 and CBS513.88 are shown in Table 2. Although there was a similar distribution of amino acids, the total amino acid pools displayed a significant difference (235 ± 8 vs 213 ± 9 µm/gDCW for DS03043 and CBS513.88, respectively). Interestingly, the pool sizes of Gln, Lys, Glu, Ala and Asp were distinctively larger than those of His, Ile, Leu, Pro, Phe and Met (p value <0.05) in both strains. It also should be noted that the pool sizes of Ala, Asp, Glu and Pro were significantly larger in *A. niger* DS03043 compared to those in *A. niger* CBS513.88 (p value <0.05). According to the original sources, the average pool size of amino acids from the family of glutamic acid was significantly higher than those of other families (Fig. 5). By comparison, the pools of histidine and aromatic amino acids were significantly decreased.

### Energy and redox metabolism

To investigate the balance of cofactor metabolism, the regeneration and consumption rates of NADPH, NADH and ATP were estimated. As displayed in Table 4, the total generation rate of NADPH for *A. niger* DS03043 and CBS513.88 were 2.06 and 1.44 mmol/gDCW h, respectively. For both strains, the amount of NADPH involved in biomass formation was remarkably higher than that in the glucoamylase production. There are mainly two sources for NADH production, including the EMP pathway and the TCA cycle. The total rates of NADH regeneration were 6.09 and 6.35 mmol/gDCW h in *A. niger* DS03043 and CBS513.88 respectively (Table 4). With a higher generation rate of NADH through the TCA cycle (4.06 mmol/gDCW h) in *A. niger* CBS513.88, the NADH from the TCA cycle was about twofold of that from EMP pathway (2.03 mmol/gDCW h). While for *A. niger* DS03043, the relatively small NADH formation rate through the TCA cycle flux (3.28 mmol/gDCW h) decreased the total generation rate of NADH. Clearly, the result further showed that in *A. niger* CBS513.88, the regeneration rate of NADH was exceeding its utilization rate.

**Table 4:** Estimated production and consumption of NADPH, NADH, ATP ( mmol/gDCW h)

Pathways	NADPH		NADH		ATP	
	*A. niger* DS03043	*A. niger* CBS513.88	*A. niger* DS03043	*A. niger* CBS513.88	*A. niger* DS03043	*A. niger* CBS513.88
Glycolysis	0	0	2.3	2.03	0.8	0.55
PP pathway	2.06	1.22	0	0	0	0
TCA cycle	0	0.22	3.28	4.06	0.49	0.79
Amino acid synthesis	–	–	0.42	0.24	–	–
Oxidative phosphorylation^a^	0	0	−5.88	−5.33	12.38	11.22
AcCoA formation by Ac	–	–	–	–	−0.1	−0.3
GA formation	−0.37	−0.06	0.09	0.02	−1.3	−0.17
Biomass formation	−1.69	−1.16	−0.54	−0.38	−8.54	−6.4

^a^The P/O ratios were set to 2.64 for mitochondrial NADH, and 1.64 for succinate and cytosolic NADH

Among the three pathways to generate ATP, including EMP, TCA cycle and the oxidative phosphorylation, the oxidative phosphorylation was the main source of ATP in both strains and the total specific generation rate of ATP were 12.38 and 11.22 mmol ATP/gDCW h respectively (Table 4) in *A. niger* DS03043 and CBS513.88. As shown in Table 4, most ATP was used for the strain growth and the fractions of ATP for biomass formation were about 68 and 57 % of total ATP generated in *A. niger* DS03043 and CBS513.88. The specific rate of ATP being used for glucoamylase synthesis was increased from 0.17 mmol ATP/gDCW h in *A. niger* CBS513.88 to 1.3 mmol ATP/gDCW h in *A. niger* DS03043. The rest ATP was potentially consumed for the non-growth maintenance requirements, which was calculated to be 3.73 and 5.68 mmol ATP/gDCW h respectively in *A. niger* DS03043 and CBS 513.88.

**Fig. 6:**
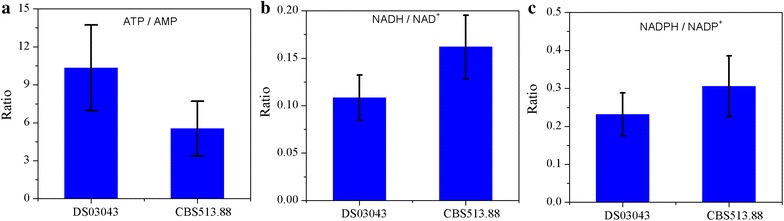
Ratios of ATP/AMP (**a**), NADH/NAD^+^ (**b**) and NADPH/NADP^+^ (**c**) in *A. niger* DS03043 and CBS513.88. The *error bars* were calculated from error propagation

As displayed in Table 3, the pool sizes of cofactors in *A. niger* DS03043 were strikingly higher than those in *A. niger* CBS513.88, especially for NAD^+^ and ATP. The higher ratio of NADH to NAD^+^ in *A. niger* CBS513.88 (Fig. 6b) was consistent with the surplus NADH supply (Table 4). By comparison, the ratio of NADPH to NADP^+^ was only slightly larger in *A. niger* CBS513.88 than DS03043 (Fig. 6c). In comparison with *A. niger* CBS513.88, the pool size of ATP within *A. niger* DS03043 was increased significantly. The energy charge $\left( {\frac{[ATP] + 0.5[ADP]}{{\left[ {ATP} \right] + \left[ {ADP} \right] + [AMP]}}} \right)$ in *A. niger* DS03043 and CBS513.88 were 0.88 ± 0.13 and 0.80 ± 0.11 respectively, consistent with the previous reported values (0.8–0.9) [20–22]. Nevertheless, the ratio of ATP to AMP was strikingly increased in *A. niger* DS03043 compared with *A. niger* CBS513.88 (Fig. 6a) and the mass spectrum profiles of ATP and AMP for two strains could be found in Additional file 4.

## Discussion

### Amino acid metabolism

The pool sizes of intracellular free amino acids are influenced by multiple factors [11], including the energy availability, the flux distribution, as well as the synthesis and degradation of specific proteins. In this work, we found that there existed no direct relations between the pool sizes of amino acids and the synthesis of glucoamylase (data not shown), except for Ala, Asp, Glu and Pro (Table 2), which may be due to the fact that during the batch phase, the main part of carbon substrate (about 80 %) was used for strain growth and aerobic respiration. Similar to previous reports [11], the overall distribution of amino acid pools were slightly affected by the enzyme production levels, indicating the robustness of strain metabolism. It has been reported that [23], for *Pichia pastoris*, the single amino acid pool size was correlated with overall cell protein amino acid compositional variations. However, in this study, we found that the amino acids pool sizes were directly related to the precursor metabolites. The average pool size of amino acids from the families of aromatic and histidine amino acids were lower on the whole (Fig. 5). Therefore, we speculated that the synthesis of these specific amino acids might be the bottleneck for further improvement in enzyme production.

### Mechanism for PP pathway regulation

**Fig. 7:**
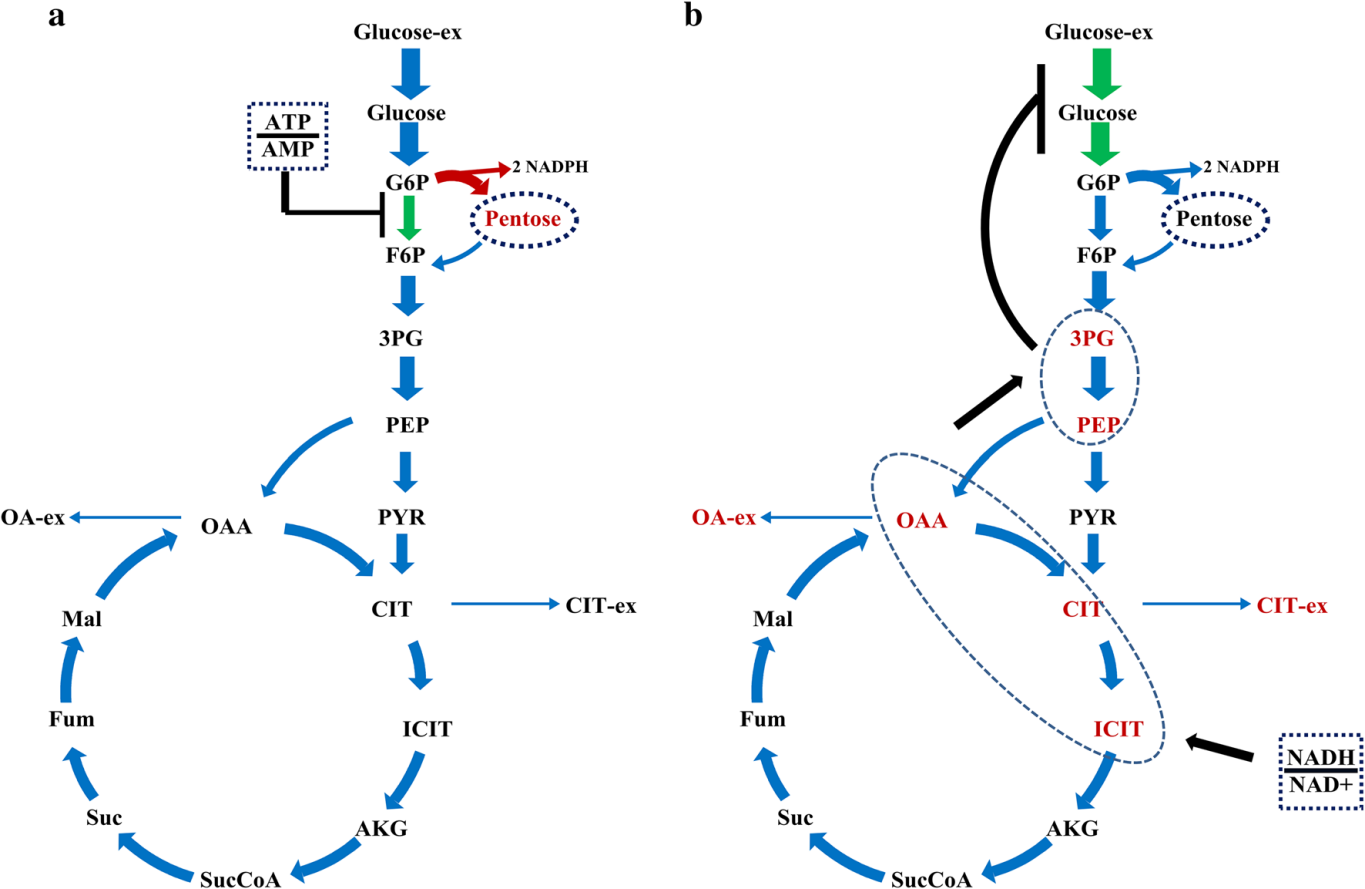
Speculated regulation mechanism revealed by integrated isotope-assisted metabolomics and ^13^C metabolic flux analysis. Possible effect of the ratio between ATP and AMP on flux redistribution in *A. niger* DS03043 (**a**) and regulation mechanism of ratio between NADH and NAD^+^ on the accumulation of OAA and PER pools, as well as on the specific glucose uptake rates in *A. niger* CBS513.88 (**b**). The *red color* in this graph represent increased flux or metabolites pool size and the *green color* represent weakened flux

To adapt to changes in the external environment or differences in genotype, the metabolic network should be flexible and, meanwhile, maintain robustness [24]. In this study, the consistent changes in metabolites pool sizes and metabolic flux demonstrate the flexibility in metabolic activities. According to Heyland et al. [25], there existed a direct correlation between the relative PP pathway flux and the yield of biomass. However, the PP pathway provides not only essential precursors for strain growth, but also the reducing power NADPH for protein synthesis. As reported previously [15, 17, 19], for strains with a high yield of protein, the flux directed into the PP pathway was always increased. *A. niger* DS03043 shows the same characteristics as the yield of glucoamylase (Y_P/S_), rather than the yield of biomass (Y_X/S_), was statistically significantly increased in *A. niger* DS03043 (Table 1). Besides the increased demand of NADPH from anabolic reaction [26], there may exist two reasons for the reinforcement of flux channeled into the PP pathway. Firstly, the key enzymes including glucose-6-phosphate dehydrogenase and 6-phosphogluconate dehydrogenase in the PP pathway may be up-regulated [27]. Secondly, the pivotal enzymes in the EMP pathway, such as glucose-6-phosphate isomerase, could be weakened to reinforce more carbon into the PP pathway [28]. Actually, the glucose-6-phosphate isomerase is inhibited by high ratio of ATP to AMP [29, 30]. In a related matter, we found that the pool size of ATP and the ratio of ATP/AMP were strikingly increased in *A. niger* DS03043 (Table 3; Fig. 6a). Thus, we hypothesize that there exists a positive correlation between the ratio of ATP/AMP and the flux through the PP pathway (Fig. 7a). However, additional evidences from multi-omics study are required to clarify the question whether the key enzymes for the EMP and PP pathways are transcriptionally regulated or not.

### TCA cycle pathway and energy metabolism

The TCA cycle pathway is the main source for NADH formation [31], as evident from the observation that the generation rate of NADH from the TCA cycle pathway was nearly twofold of that from the EMP pathway (Table 4). Via oxidative phosphorylation, NADH was transformed into ATP to meet the energy demand for maintenance, growth and product formation. For protein synthesis, the changes in the TCA cycle flux might depend on the strain. For *Schizosaccharomyces pombe* [16], it has been reported that to compensate for metabolic burden for heterologous protein synthesis, the flux through TCA cycle pathway was increased. As a result, there exists a positive correlation between the protein yield and the flux directed into the TCA cycle pathway. In contrast, for *A. niger* producing fructofuranosidase [15], the flux of the TCA cycle pathway dropped. Similarly, we found that this flux changed in the same direction in *A. niger* DS03043 (Fig. 3) and, more strikingly, the pool sizes of TCA cycle intermediates, like OAA, CIT, is CIT and AKG were also reduced (Fig. 4), which was accompanied with the reduction in by-products (oxalic acid and citric acid) formation (Table 1). The reported m_ATP for *A. niger* was 1.9 mmol ATP/gDCW h [32], while the calculated maintenance energy for both strains in this study were obviously higher, especially for *A. niger* CBS513.88. In general, the ATP cost for non-growth maintenance depends on product and by-products formation and the type of substrate used. It has been reported [33] that the cellular maintenance ATP consumption in engineered *E. coli* cells producing fatty acids was nearly twofold of that in control strain due to an increased osmotic stress resulting from product accumulation. Chung and co-workers [34] also reported that maintenance requirements for *P. pastoris* growing on glucose and glycerol–methanol mixed media were 2.3 and about 6 mmol ATP/gDCW h, respectively. Moreover, the transmembrane transport of organic acids was also accompanied with loss of energy [35]. Therefore, it could be concluded here that *A. niger* CBS513.88 needs more non-growth maintenance energy due to the accumulation and secretion of oxalic and citric acids, which, to some extent, decreased the utilization efficiency of energy in *A. niger* CBS513.88.

### How metabolites modulate metabolic flux ?

Among thousands of metabolites, cofactors participate in 300 oxidation and reduction reactions [36] and play an important role in regulating the cell metabolic activities. Generally, the formation of by-products are correlated with the cofactors metabolism, for example, the glycerol synthesis could contribute to the recycle of NADH [37]. It has been reported that the substrate uptake rate was depressed under higher intracellular ratio of NADH/NAD^+^ [38]. However, the mechanisms for the modulation in fluxes via cofactors are generally not clear. The physiological parameters of *A. niger* CBS513.88 and DS03043, as found in this study, indicated that with large amounts of by-products formation the specific growth rate of the wild type strain was decreased. Interestingly, we found that the ratios of NADH/NAD^+^ and NADPH/NADP^+^ in *A. niger* CBS513.88 were higher than that in *A. niger* DS03043 (Fig. 6b, c), meaning that the *A. niger* CBS513.88 was in high redox condition. Accompanied with the high ratio of NADH/NAD^+^, the intermediates in the TCA cycle (CIT, is CIT, AKG and OAA) were accumulated in *A. niger* CBS513.88 (Fig. 4). The accumulation of organic acid from the TCA cycle also happened under oxygen limited conditions when the NADH recycle was hindered [39]. As reported by Heux et al. [38] and Hou et al. [40], decreasing the intracellular NADH level by introducing NADH oxidase or alternative oxidase lowered the by-products formation greatly. So it could be speculated that the higher ratio of NADH/NAD^+^ contributes to the increase in pool sizes of these intermediates from TCA cycle. As PEP was linked with the TCA cycle through anaplerotic reactions, the pool size of PEP in the cytoplasm was also obviously increased accordingly in *A. niger* CBS513.88 (Fig. 4).

According to Ogawa et al. [41], the accumulation of PEP could regulate the glucose uptake and glycolytic flux in cells by inhibiting glucokinase and glucose-6-phosphate isomerase. For mammalian cells culture, it was found that the high lactate level can exert an inhibitory effect on glycolysis enzyme phosphofructokinase, thus reducing the glycolytic flux [42]. So for *A. niger* CBS513.88 in this work, the lower absolute glycolytic flux (Fig. 4) might be caused by the inhibitory effect of organic acid, including PEP and 3PG, which in turn results in a lower specific growth rate (Table 1). As the accumulation of organic acids is related to the cell redox condition, the regulation of glycolytic flux actually seems to depend on the redox condition of the strain. In a comprehensive view, the possible regulation mechanism in flux and metabolites pools by the ratio of NADH/NAD^+^ could be summarized by Fig. 7b. With integrative analysis of metabolomics and ^13^C MFA, this work provides new clues to illustrate the interactive effects of flux and metabolites, which could be applied in other strains or process studies as well.

## Conclusion

In this study, the combination of ^13^C metabolic flux analysis and quantitative metabolomics was adopted for the first time to investigate the metabolic changes in *A. niger* correlated with high glucoamylase production, initially revealing the metabolic regulation mechanisms between intracellular flux redistribution and metabolites pool sizes. The consistency in ^13^C metabolic flux analysis and metabolites quantification indicated that an imbalance of NADH formation and consumption led to the accumulation and secretion of organic acids in *A. niger* CBS513.88. The significantly reduced by-products formation rates resulted in an increased energy utilization efficiency and specific growth rate in *A. niger* DS03043. To enable a more efficient enzyme production and faster cell growth, the total amino acids pool size, the ratio of ATP to AMP and the flux channeled into the PP pathway were obviously improved in *A. niger* DS03043, which bring new insights on metabolic engineering targets for improved enzyme production using *A. niger*.

## Methods

### Strain and cultivations

Two *A. niger* strains with different glucoamylase production levels were kindly donated by DSM Corporation (Delft, the Netherlands). *A. niger* DS03043, a high glucoamylase-producing strain, contains seven copies of glucoamylase *gla*A gene, while *A. niger* CBS513.88, as a wild-type parent strain, contains only one *gla*A gene copy [43].

To obtain spores, Petri dishes containing PDA (Potato Dextrose Agar) medium were incubated with spores from a frozen stock (stored in 50 % glycerin at −80 °C). The seed cultivation was conducted in 500 mL shaking flasks with three baffles. Each flask was inoculated with 10^7^ spores per 100 mL broth. The batch cultivations were conducted in a 5 L fermenter with an electronic balance, inoculated with the seed broth from the shake flasks after 24 h seed culture at 34 °C, with the speed of shaking table controlled at 150 rpm. The working volume for 5 L fermenter was 3.0 L. The agitation and aeration in the 5 L fermenter were kept at 375 rpm and 1 vvm respectively, with overpressure maintained at 0.05 MPa and temperature at 34 °C during the whole process. The pH was automatically maintained at pH 4.5 with 5 % (w/w) NH_4_·OH. The cultivations for each strain were performed in three independent cultivation replicates.

The medium for seed culture contained (g/L) glucose·H_2_O 22 and spray dried corn steep liquor 20. Before sterilization, the initial pH was adjusted to 6.5 using 3 mol/L NaOH. A chemically modified defined medium was used for all cultivations in this study, which contained (g/L) glucose·H_2_O 20, KH_2_PO_4_ 3, NaH_2_PO_4_·H_2_O 1.5, (NH_4_)_2_SO_4_ 3, MgSO_4_·7H_2_O 1.0, CaCl_2_·2H_2_O 0.1, ZnCl_2_ 0.02, CuSO_4_·5H_2_O 0.015, CoCl_2_·6H_2_O 0.015, MnSO_4_·H_2_O 0.04, FeSO_4_·7H_2_O 0.3.

### ^13^C labeling experiments

Considering the high cost, the ^13^C labeling experiments were conducted in a reactor system consisting of a 5 L bioreactor and a 250 mL mini bioreactor equipped with pH and oxygen sensors. Before the formal experiment, the agitation and aeration in the mini bioreactor were carefully optimized to guarantee that the strain had similar physiological parameters in two scales of fermenters. The initial glucose concentration in the 5 L bioreactor was 10 g/L. When the glucose concentration decreased to approximately 5 g/L, about 170 mL broth was rapidly transferred from the 5 L bioreactor into the mini bioreactor. Then the labeled glucose including 0.56 g [U–^13^C]–Glc and 1.41 g [1–^13^C]–Glc (isotopic enrichment 98–99 %, Cambridge Isotope Laboratories, Inc.) were added as a pulse into the mini bioreactor.

### Quantification of biomass and enzyme activity

10 mL broth was filtered through pre-dried and pre-weighed suction filter paper. Before filtering, the filter should be dried to a constant weight (24 h at 80 °C). To remove solutes, the samples were rinsed three times with deionized water. Then the wet filters with the biomass were put in the 80 °C oven and dried for 24 h. The dried filter was re-weighed immediately. A previous protocol was adopted to determine the enzyme activity in all samples [44].

### Analysis of extracellular glucose and organic acids

A glucose analyzer (Shandong Academy of Sciences, China) was used for residual glucose concentration analysis. The amounts of extracellular organic acids (acetic, citric, oxalic, fumaric, malic, pyruvic, and succinic acids) were analyzed using a high-performance liquid chromatography (HPLC) system. An ion-exclusion column (Hi-Plex H, Agilent) was eluted at 50 °C with 10 mM H_2_SO_4_ at a flow rate of 0.5 mL/min and connected with an absorbance detector spectrophotometer at 210 nm. No acetic acid was detected under these conditions of cultivation.

### Sampling and quantification of intracellular metabolites concentration

The protocol for quantification of intracellular metabolites pool size was adapted from the works of Douma et al. [45]. With a dedicated fast-sample equipment, 1–2 mL broth was rapidly withdrawn from sensor bioreactors in tubes with 10 mL precooled quench solution (−27.6 °C 40 % v/v methanol solution) at 30, 60, 90, 120, 180, 240 and 300 min since the start of ^13^C labelled experiments. The tubes were weighed before and after sampling procedure in order to estimate the exact amount of broth taken. Subsequently, fast filtration with a vacuum pump was conducted to remove the extracellular metabolites as soon as possible. Then, 120 mL precooled quench solution was used to wash the filter cake. To accurately determine the metabolites concentration, Isotope Dilution Mass Spectrometry (IDMS) [46] was adopted in this study. During the extraction process, the washed filter cake and ^13^C internal standard solution were together added into the pre-warmed 25 mL 75 % (v/v) ethanol solution and the extraction continued for 3 min at 95 °C. The metabolites pools were determined with the UPLC-MS/MS (Thermo Fisher Scientific Coporation).

For analysis of the intracellular pool size of NADH, NAD^+^, NADPH, and NADP^+^, the EnzyFluo™ Assay Kit (BioAssay Systems Coporation) was used. Firstly, 1 mL broth was quenched with 10 mL quench solution. The supernatant was discarded after centrifugation at −4 °C and 6000 rpm. The remaining processes were referred to the standard specifications (BioAssay Systems Coporation).

### Analysis of amino acids enrichment with GC–MS

To obtain the enrichment of intracellular free amino acids during the ^13^C labelled experiments, the extracting solution was concentrated to 100 μL of supernatant before being transferred to a glass vial. After lyophilization, 80 μL acetonitrile and 80 μL of *N*-methyl-*N*-(tert-butyldimethylsilyl) trifluoroacetamide (MTBSTFA, Thermo Scientific) were added and the vial was incubated for 2 h at 60 °C. After that, the sample was centrifuged (10,000*g*, 2 min) and 160 μL of the supernatant was transferred to a GC glass vial with an insert. The sample was then analyzed by GC–MS instrument coupled to a 5975 C MSD single quadrupole mass spectrometer (Agilent, Santa Clara, CA, USA).

### Calculation of metabolic fluxes based on ^13^C-labeled experiments

^13^C-MFA was performed using isotopomer Network Compartmental Analysis (INCA) [18], which is based on the elementary metabolite units (EMU) framework [47]. The *A. niger* network model used for flux calculations was described previously [15] and is given in Additional file 5. In brief, the model included all major reactions of the central carbon metabolism, amino acid biosynthesis, and lumped biomass formation. To determine the goodness-of- fit, the ^13^C-MFA fitting results were subjected to a χ^2^-statistical test [48]. The estimated fluxes were considered acceptable only when the obtained minimal weighted residual was below the χ^2^ at a 95 % confidence level. At convergence, accurate 68 and 95 % confidence intervals were computed for all estimated fluxes by evaluating the sensitivity of the minimized SSR to flux variations. The precision (or standard errors) of estimated fluxes were determined as follows [48]:

$${\text{Flux precision }}\left( {\text{stdev}} \right) \, = \, \left[ {\left( {{\text{flux}}_{{{\text{upper bound 95}}\% }} } \right) \, {-} \, \left( {{\text{flux}}_{{{\text{lower bound 95}}\% }} } \right)} \right]/ 4$$

## Additional files

**Additional file 1**. The measured and estimated labelling information of amino acids fragments for optimal flux distributions.
**Additional file 2**. The best-fit flux distribution and minimal weighted residual from the parameter estimations.
**Additional file 3**. Overview of the current status of flux analysis in different *Aspergillus* species.
**Additional file 4**. Mass spectrum profiles of AXP for two strains and standard curves of AXP with ^13^C labelled metabolites as internal standards.
**Additional file 5**. Stoichiometric metabolic model of *Aspergillus niger*.
