# Supplementary material for: Integrated isotope-assisted metabolomics and 13C metabolic flux analysis reveals metabolic flux redistribution for high glucoamylase production by Aspergillus niger
Source: Microb Cell Fact. 2015 Sep 17;14:147. doi: 10.1186/s12934-015-0329-y (PMC4574132; doi:10.1186/s12934-015-0329-y)
Supplement: Supplementary file 3 — Additional file 3. Overview of the current status of flux analysis in different Aspergillus species. [file 12934_2015_329_MOESM3_ESM.pdf]

Overview of the current status of flux analysis in different *Aspergillus* species.

Reference	Pedersen et al. 1999	Pederson et al. 2000	David et al. 2005	Anderson et al. 2008	Meijer et al. 2009	Panagiotou et al. 2009	This work
Strain	*A. oryzae*	*A. niger*	*A. nidulans*	*A. niger*	*A. niger*	*A. nidulans*	
	A1560 /*CF1.1*	BO-1/ Δ *oah 4-1*	A187/*creA*	*wt/i*MA871	N402/*icL*	*wt/msaS/msaS+xpkA*	wt/producer
**Enzyme**							
glucose uptake	100/100	100/100	100/100	100/100	100/100	100/100 /100	100/100
***hxk*, hexokinase**	100/100	100/100	100/100	100/100	100/100	100/100 /100	100/100
***g6pdh*, 6-phosphogluconate dehydrogenase**	35/40	58/67	78/61	46/48	95/76	57/46/52	41/56
***pgi*, glucose-6-phosphate isomerise**	48/43	30/20	---/---	39/35	05/20	28/39/36	48/32
***pfk*, 6-phosphofructokinase**	67/63	61/51	56/57	55/53	67/70	39/50/40	66/58
***tk1*, transketolase**	20/23	35/41	26/22	25/27	62/51	15/09/04	24/33
***tk2/tal*, transketolase/ transaldolase**	9/8	16/109	26/22	20/21	62/51	15/09/04	15/20
***man*$_{cycle}$, mannitol cycle**	2/3	---/---	0/0	---/---	0/0	---/---/---	---/---
***glo*$_{ycle}$, glycerol cycle**	1/1	---/---	5/1	---/---	0/0	---/--/---	---/---
***pgk*, phosphoglycerate kinase**	142/140	130/120	122/125	123/118	156/166	110/116/115	138/125
***ser from gly*, c1-Metabolism**	---/---	---/---	3/3	---/---	1/3	---/---/---	0/0
***gly from ser*, c1-Metabolism**	---/---	---/---	0/0	---/---	0/4	---/---/---	3/4
***pyrk*, pyruvate kinase**	129/119	103/95	111/115	108/106	154/163	110/116/115	65/77
***pyrc*, pyruvate carboxylase**	18/21	27/25	23/22	15/ 12	8/34	23/22/19	56/25
***pepck*, phosphoenolpyruvate carboxykinase**	---/---	---/---	---/---	---/---	---/---	---/---/---	---/---
***oaah*, oxaloacetate hydrolase**	---/---	3/0	---/---	3/0	0/0	---/---/---	21/5
***me*, malic enzyme**	---/---	---/---	---/---	---/---	---/---	---/---/---	15/0
***pyrdh*, pyruvate dehydrogenase**	97/81	103/95	69/79	108/106	146/101	71/77/82	68/60
***cs/ac*, citrate lyase/acetate-CoA ligase**	93/76	82/74	52/58	78/77	200/184	83/75/79	61/36
***ictdh*, isocitrate dehydrogenase**	70/54	64/52	52/58	45/38	153/106	83/75/79	61/36
***ogadh*, α-ketoglutarate dehadrogenase**	52/33	54/39	52/58	45/38	153/106	79/79/94	54/27
***sucdh*, succinate dehadrogenase**	59/43	54/39	39/47	38/31	153/106	79/79/94	54/24
***fum*, fumarase**	59/43	54/39	39/47	38/31	153/106	79/79/94	55/28
***mdh*, malate dehydrogenase**	79/60	54/39	39/47	38/31	153/106	79/79/94	40/28

*The data (except from this work) in above table was summarized by Wittmann et al[1].

1. Driouch, H., Melzer, G. & Wittmann, C. Integration of in vivo and in silico metabolic fluxes for improvement of recombinant protein production. *Metab. Eng.* **14**, 47-58 (2012).
