# Supplementary material for: Integrated isotope-assisted metabolomics and 13C metabolic flux analysis reveals metabolic flux redistribution for high glucoamylase production by Aspergillus niger
Source: Microb Cell Fact. 2015 Sep 17;14:147. doi: 10.1186/s12934-015-0329-y (PMC4574132; doi:10.1186/s12934-015-0329-y)
Supplement: Supplementary file 4 — Additional file 4. Mass spectrum profiles of AXP for two strains and standard curves of AXP with 13C labelled metabolites as internal standards. [file 12934_2015_329_MOESM4_ESM.docx]

Mass spectrum profiles of AXP for two strains

*A.niger* DS03043


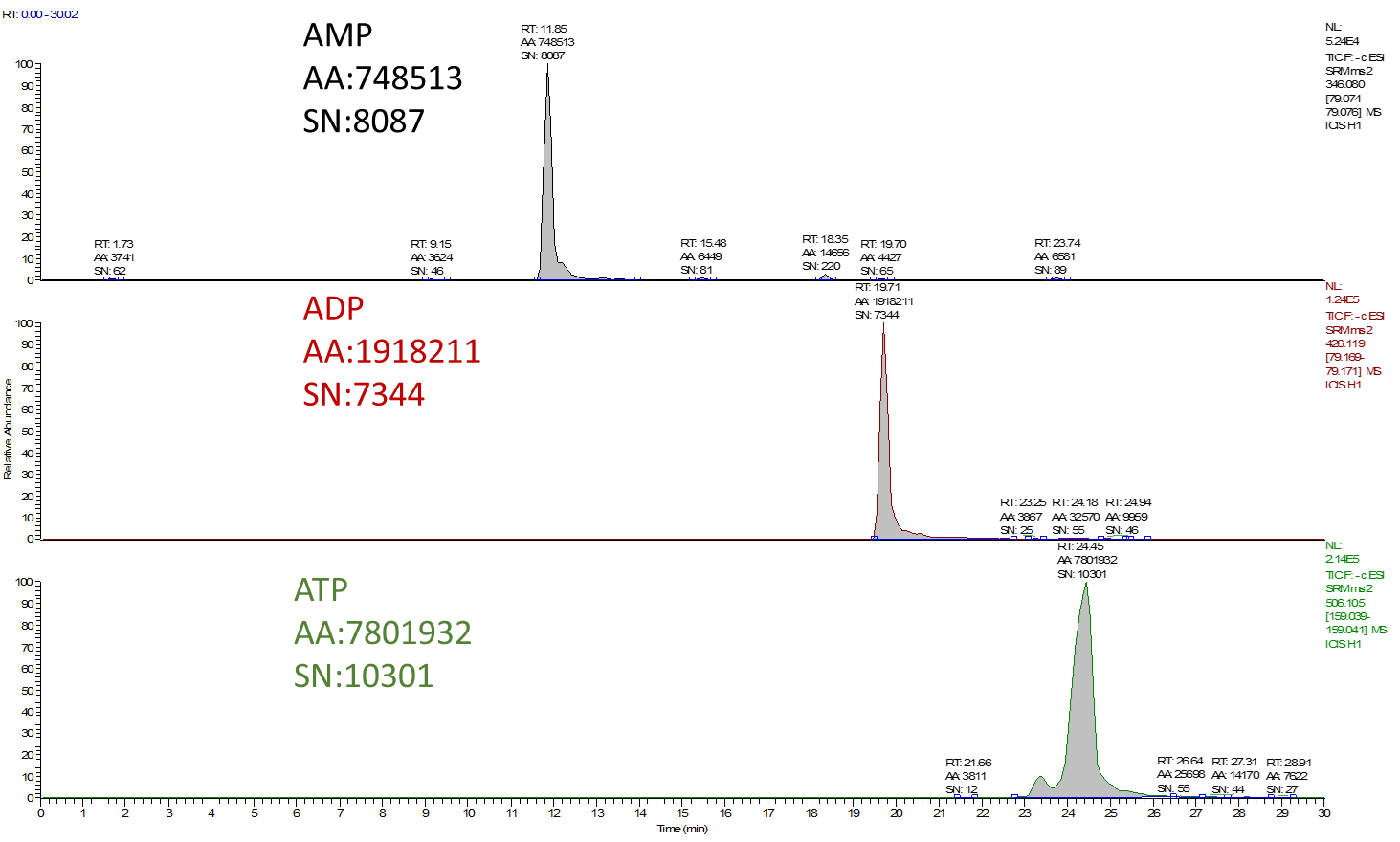


*A.niger* CBS513.88


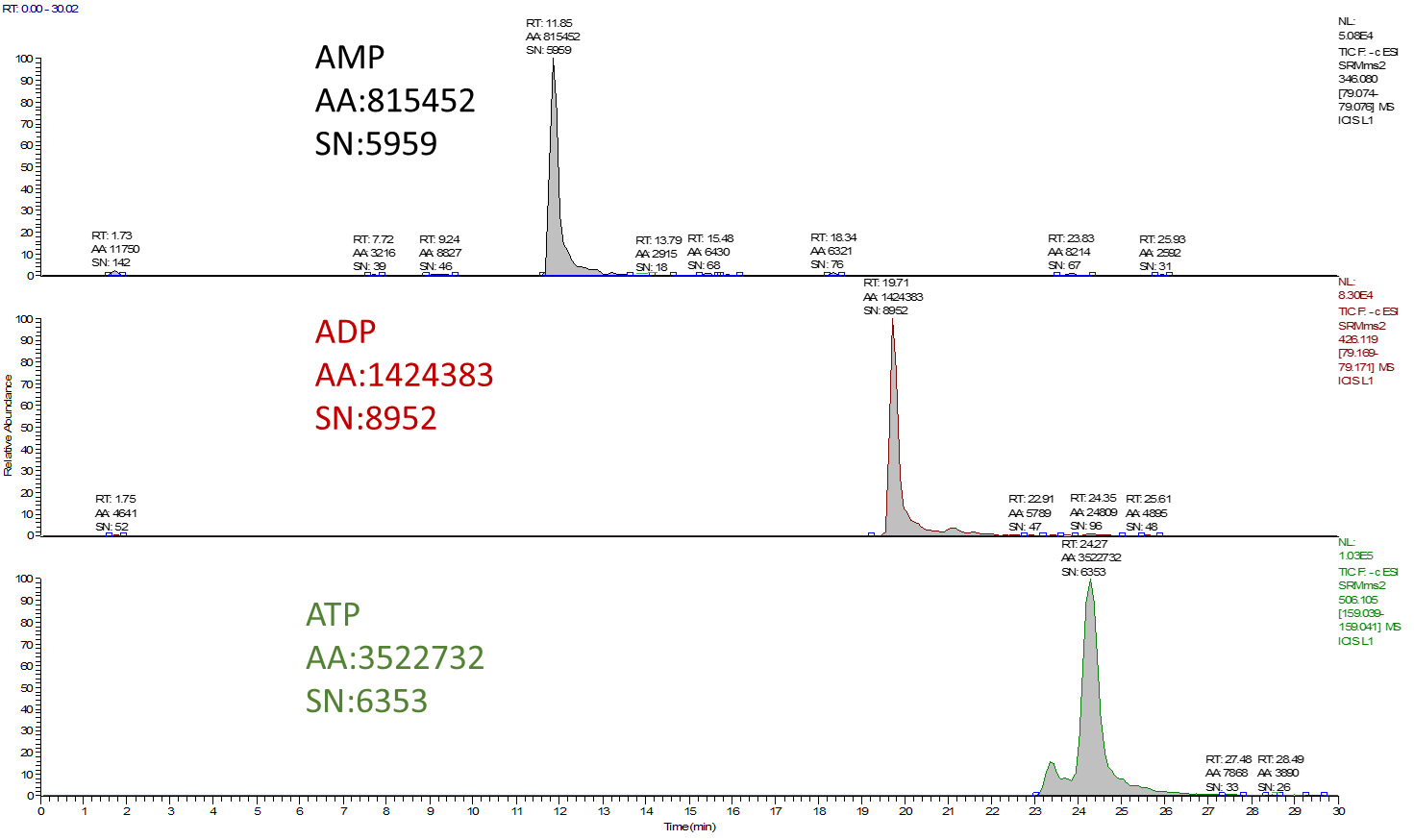


Note: AA-area; SN-signal to noise ratio

Standard curves of AMP, ADP, ATP with ^13^C labelled metabolites as internal standards
