# Supplementary material for: Integrated isotope-assisted metabolomics and 13C metabolic flux analysis reveals metabolic flux redistribution for high glucoamylase production by Aspergillus niger
Source: Microb Cell Fact. 2015 Sep 17;14:147. doi: 10.1186/s12934-015-0329-y (PMC4574132; doi:10.1186/s12934-015-0329-y)
Supplement: Supplementary file 5 — Additional file 5. Stoichiometric metabolic model of Aspergillus niger. [file 12934_2015_329_MOESM5_ESM.pdf]

Stoichiometric metabolic model of *Aspergillus niger*

1. Metabolic model of *A .niger*

Substrate uptake
GLC.ext (abcdef) -> GLC (abcdef)
O2.ext -> O2
NH3.ext -> NH3
SO4.ext -> SO4

Glycolysis and PP Pathway
GLC (abcdef) + ATP -> G6P (abcdef)
G6P (abcdef) <-> F6P (abcdef)
F6P (abcdef) + ATP -> FBP (abcdef)
FBP (abcdef) <-> DHAP (cba) + GAP (def)
DHAP (abc) <-> GAP (abc)
GAP (abc) <-> 3PG (abc) + ATP + NADH
3PG (abc) <-> PEP (abc)
PEP (abc) -> Pyr (abc) + ATP
G6P (abcdef) -> 6PG (abcdef) + NADPH
6PG (abcdef) -> Ru5P (bcdef) + CO2 (a) + NADPH
Ru5P (abcde) <-> X5P (abcde)
Ru5P (abcde) <-> R5P (abcde)
X5P (abcde) <-> GAP (cde) + EC2 (ab)
F6P (abcdef) <-> E4P (cdef) + EC2 (ab)
S7P (abcdefg) <-> R5P (cdefg) + EC2 (ab)
F6P (abcdef) <-> GAP (def) + EC3 (abc)
S7P (abcdefg) <-> E4P (defg) + EC3 (abc)

Specialties around G3P and C1-Metabolism
3PG (abc) + Glu (defgh) -> Ser (abc) + AKG (defgh) + NADH
Ser (abc) <-> Gly (ab) + MEETHF (c)
Gly (ab) <-> CO2 (a) + MEETHF (b) + NADH + NH3

TCA Cycle
Pyr.m (abc) -> AcCoA.m (bc) + CO2 (a) + NADH
OAA.m (abcd) + AcCoA.m (ef) -> CIT.m (dcbfea)
CIT.m (abcdef) <-> ICit (abcdef)
ICit (abcdef) <-> AKG (abcde) + CO2 (f) + NADH
AKG (abcde) -> SucCoA (bcde) + CO2 (a) + NADH
SucCoA (abcd) <-> Suc (abcd) + ATP
Suc (abcd) <-> Fum (abcd) + NADH
Fum (abcd) <-> Mal (abcd)
Mal (abcd) <-> OAA.m (abcd) + NADH

Mal (abcd) -> Pyr.m (abc) + CO2 (d) + NADPH

ANAPL Pathways
PEP (abc) + CO2 (d) <-> OAA (abcd)
OAA (abcd) + ATP -> PEP (abc) + CO2 (d)

Oxalate Formation
OAA (abcd) -> Ac (ab) + OA (cd)

Formation of AcCoA in the Cytosol
Ac (ab) + ATP -> AcCoA (ab)
CIT (abcdef) + ATP -> AcCoA (bc) + OAA (adef)

Transport Reactions Cytosol-Mitochondria
CIT.m (abcdef) -> CIT (abcdef)
Pyr (abc) -> Pyr.m (abc)
AcCoA (ab) <-> AcCoA.m (ab)
OAA.m (abcd) <-> OAA (abcd)

Amino Acid Biosynthesis
AKG (abcde) + NADPH + NH3 -> Glu (abcde)
Glu (abcde) + ATP + NH3 -> Gln (abcde)
Glu (abcde) + ATP + 2*NADPH -> Pro (abcde)
Glu (abcde) + AcCoA.m (kl) + 2*ATP + 4*NADPH -> Lys (abcdek) + CO2 (l)
Glu (abcde) + CO2 (f) + Gln (ghijk) + Asp (lmno) + AcCoA.m (pq) + 5*ATP + NADPH-> Arg (abcdef) + AKG (ghijk) + Fum (lmno) + Ac (pq)
OAA (abcd) + Glu (efghi) -> Asp (abcd) + AKG (efghi)
Asp (abcd) + 2*ATP + NH3 -> Asn (abcd)
Pyr.m (abc) + Glu (defgh) -> Ala (abc) + AKG (defgh)
Thr (abcd) -> Gly (ab) + AcCoA (cd) + NADH
Ser (abc) + AcCoA (de) + 3*ATP + 4*NADPH + SO4 -> Cys (abc) + Ac (de)
Asp (abcd) + 2*ATP + 2*NADPH -> Thr (abcd)
Asp (abcd) + METHF (e) + Cys (fgh) + SucCoA (ijkl) + ATP + 2*NADPH -> Met (abcde) + Pyr (fgh) + Suc (ijkl) + NH3
Pyr.m (abc) + Pyr.m (def) + Glu (ghijk) + NADPH -> Val (abcef) + CO2 (d) + AKG (ghijk)
AcCoA.m (ab) + Pyr.m (cde) + Pyr.m (fgh) + Glu (ijklm) + NADPH -> Leu (abdghe) + CO2 (c) + CO2 (f) + AKG (ijklm) + NADH
Thr (abcd) + Pyr (efg) + Glu (hijkl) + NADPH -> Ile (abfcdg) + CO2 (e) + AKG (hijkl) + NH3
PEP (abc) + PEP (def) + E4P (ghij) + Glu (klmno) + ATP + NADPH -> Phe (abcefghij) + CO2 (d) + AKG (klmno)
PEP (abc) + PEP (def) + E4P (ghij) + Glu (klmno) + ATP + NADPH -> Tyr (abcefghij) + CO2 (d) + AKG (klmno) + NADH

Ser (abc) + R5P (defgh) + PEP (ijk) + E4P (lmno) + PEP (pqr) + Gln (stuvw) + 3*ATP + NADPH -> Trp (abcedklmnoj) + CO2 (i) + GAP (fgh) + Pyr (pqr) + Glu (stuvw)
R5P (abcde) + FTHF (f) + Gln (ghijk) + Asp (lmno) + 5*ATP -> His (edcbaf) + AKG (ghijk) + Fum (lmno) + 2*NADH

Transhydrogenase reaction
MEETHF (a) + NADH -> METHF (a)
MEETHF (a) -> FTHF (a) + NADPH

Polyol Metabolism
F6P (abcdef) + NADH -> MAN (abcdef)
DHAP (abc) + NADH -> GLO (abc)
E4P (abcd) + NADH -> EOL (abcd)

Secretion to the Medium
Ac (ab) -> Ac.ext (ab)
OA (ab) -> OA.ext (ab)
CIT (abcdef) -> CIT.ext (abcdef)
MAN (abcdef) -> MAN.ext (abcdef)
GLO (abc) -> GLO.ext (abc)
EOL (abcd) -> EOL.ext (abcd)
CO2 (a) -> CO2.ext (a)
GA -> GA.ext

Biomass synthesis reaction (mM/g biomass)
0.275*Ala + 0.11*Arg + 0.056*Asn + 0.168*Asp + 0.021*Cys + 0.24*Glu + 0.08*Gln + 1.093*Gly + 0.055*His + 0.106*Ile + 0.188*Leu + 0.173*Lys + 0.028*Met + 0.083*Phe + 0.118*Pro + 0.796*Ser + 0.134*Thr + 0.034*Trp + 0.055*Tyr + 0.143*Val + 1.6*G6P + 0.86*F6P + 0.281*R5P + 0.213*MAN + 0.6*GLO + 0.5*EOL + 0.188*E4P + 0.08*GAP + 0.477*3PG + 0.306*PEP + 0.866*Pyr + 3.328*AcCoA + 0.15*Ac + 0.538*OAA + 0.41*AKG + 8.125*NADPH + 0.33*O2 + 3.86*NADH + 0.00281*CO2 + 61*ATP -> biomass

Glucoamylase synthesis reaction (mM/mM GA)
65*Ala + 20*Arg + 25*Asn + 44*Asp + 10*Cys + 25*Glu + 17*Gln + 46*Gly + 4*His + 24*Ile + 48*Leu + 13*Lys + 3*Met + 22*Phe + 22*Pro + 88*Ser + 74*Thr + 20*Trp + 27*Tyr + 42*Val + 2554*ATP -> GA

Dilution reaction
CO2.unlabeled (a) + CO2 (b) -> CO2 (a) + CO2.out (b)
Thr <-> Thr.p + dummy1
Val <-> Val.p + dummy2
Ser <-> Ser.p + dummy3

2. Unbalanced metabolites

Ac.ex, OA.ext, CIT.ext, MAN.ext, GLO.ext, EOL.ext, GA.ex, ADP, ATP, Biomass, CO2.unlabeled, CO2.out, GLC.ex, NAD(H), NAD(P)H

3. Abbreviations

2PG	2-phospho-D-glycerate
3PG	3-phosphoglycerate
3PGP	3-phospho-D-glyceroyl phosphate
Aaa	α-aminoadipic acid
AC	acetate
AcCoA	acetyl-CoA
AKG	α-Ketoglutaric acid
Ala	alanine
Arg	arginine
Asn	asparagine
Asp	aspartate
CIT	citrate
CO2	carbon dioxide
DHAP	dihydroxyacetone-phosphate
E4P	erythrose-4-phosphate
EOL	erytrithol
F6P	fructose-6-phosphate
FBP	1,6 Fructose Diphosphate
FUM	fumarate
FUM	fumarase
G6P	glucose-6-phosphate
GA	glucoamylase
GAP	glyceraldehyde-3-phosphate
GLC	glucose
Gln	glutamine
GLO	glycerol
Glu	glutamate
His	histidine
ICT	isocitrate
Ile	isoleucine
Leu	leucine
Lys	lysine
Mal	malate
MAN	mannitol
Met	methionine
OA	oxalate
OAA	cytosolic oxaloacetate
OGA	α-ketoglutarate
Orn	ornithine

P5P	pentose-5-phosphate
PEP	phosphoenolpyruvate
Phe	phenylalanine
Pro	proline
PYR	pyruvate
R5P	ribose-5-phosphate
Ru5P	ribulose 5-phosphate
S7P	sedoheptulose-7-phosphate
Ser	serine
SUC	succinate
Thr	threonine
Trp	tryptophan
Tyr	tyrosine
Val	valine
X5P	D-Xylulose 5-phosphate
